# Comparing the effect of Glucosamine and Glucosamine With Alendronate in Symptomatic Relieve of Degenerative Knee Joint Disease: A Double- blind Randomized Clinical Trial Study

**Published:** 2012-08-25

**Authors:** Hamid Reza Arti, Mohammad Ebrahim Azemi

**Affiliations:** 1 Department of Orthopedic Surgery School of Medicine Ahvaz Jundishapur University of Medical Sciences, Ahvaz, IR Iran; 2 Department of Pharmacognosy, Ahvaz Jundishapur University of Medical Sciences, Ahvaz, IR Iran; 3 Medical Herbs and Natural Products Research Center, Jundishapur University of Medical Sciences, Ahvaz, IR Iran

**Keywords:** Alendronate, Glucosamine, Osteoarthritis, Clinical Trial

## Abstract

**Background:**

Degenerative Joint Disease (DJD) is the most common joint disease in human beings. Previous studies have explained that glucosamine is preferred as placebo and in efficacy compared with NSAID’s in treatment of patients’ knee osteoarthritis. Alendronate was used to treat osteoporotic patients and its efficacy was established.

**Objectives:**

The aim of this study was to compare the efficacy of administration of glucosamine alone and its combination with alendronate in osteoarthritis of the knee.

**Patients and Methods:**

The study included 130 patients with osteoarthritis who randomly received glucosamine alone (group II) (500mg TDS), or combination of glucosamine (500mg TDS) and alendronate (70mg weekly) (group I) for 12 weeks. Patients were evaluated on 1, 3, 6 and 12 weeks after beginning the treatment to evaluate efficacy of each treatment.

**Results:**

Statistically, there was no significant difference in pain index (P > 0.05) but in the two groups the mean of pain index decreased in a similar fashion. The stiffness index in combination treatment group (group I) decreased more than glucosamine group (group II) (P < 0.05). The function of joints in combination treatment group (group I) improved after 12weeks. The bone mineral density (BMD) at 12weeks in combination therapy group improved.

**Conclusions:**

Combination therapy of glucosamine and alendronate indicated significant improvement of stiffness, function, BMD of osteoarthritis compared with glucosamine alone but there was no statistically significant decrease in pain index. It can be concluded that the combination of glucosamine and alendronate provide better and more rapid improvement in patients with osteoarthritis.

## 1. Background

Degenerative joint disease or osteoarthritis is the most common destructive joint disease in human beings and also the most common cause of chronic disability among elderly people in developed countries. The biggest risk factor for developing osteoarthritis is age. Severe and frequent use of knee joints , trauma , and obesity are other important risk factors in progressing knee osteoarthritis (1). The prevalence of knee osteoarthritis is between 1.4% - 20% in different countries and in Iran it is 15.3% to 19.3% in urban and rural areas respectively and female to male ratio is about 2:1. The prevalence of symptomatic knee osteoarthritis is 4.9% among adults aged more than 26 and 12.1% among adults aged more than 60 years old (2). Normal articular cartilage is composed of chondrocytes (about 5% of its normal tissue) and the extracellular matrix, (about 95% of its normal tissue) (1). Articular cartilage changes are seen in osteoarthritis results from cartilage matrix macromolecules reaction between the matrix and the chondrocytes producing and supporting matrix (3).

Diagnosis of disease is based on clinical and radiographic evidences. In the early stages of the disease, radiographs may be normal, but with progression of disease radiographic findings will appear and include sclerosis of the subchondral bone, appearance of subchondral cysts, marginal osteophytes, joint space narrowing in one side and widening in the other side of joint (1). The aims of osteoarthritis treatment are diminishing pain, maintaining joint mobility and minimizing disability of persons. Treatment should be determined based on the status of each patient separately.

For mild cases, taking care of the patients and protecting the joints and sometimes administration of anti inflammatory analgesic is sufficient. The main stay of Pharmacological treatment of osteoarthritis is reduction of pain. Most non-steroidal anti-inflammatory drugs (NSAID’s) reduce joint pain and improve its mobility. Glucocorticoids as topical or intra-articular drugs may cause significant improvement in symptoms for weeks to months, but they are not administered as a systemic drug for treatment of osteoarthritis (4). Another treatment method of osteoarthritis is to administer protective cartilage agents such as glucosamine.

## 2. Objectives

The aim of this study was to compare the effects of glucosamine alone and combination of glucosamine and alendronate administration to treat knee osteoarthritis. According to the high prevalence of osteoarthritis, especially knee osteoarthritis ,and treatment costs of the disease for the health care system, identification of effective treatment methods is an important factor in medical treatment of knee osteoarthritis. As in elderly patients osteoarthritis and osteoporosis may exist together simultaneously, these two diseases may be influenced by passing the time.

Glucosamine is a useful drug in osteoarthritis treatment and alendronate is considered as one of the drugs used to treat osteoporosis. In osteoarthritis, treatment emphasizes on articular cartilage protection and repair, however, advanced osteoporosis is often associated with the formation of bone cysts and bone loss of subchondral bone. Administration of alendronate and glucosamine combination for simultaneous protection of cartilage and bone may have therapeutic benefits. The current study aimed to test the hypothesis that combination administration of glucosamine and alendronate was more useful than administration of glucosamine alone. According to the literature review no previous study had been done on this issue.

## 3. Patients and Methods

This study was a prospective double-blind randomized clinical trial study. The population under study were patients who referred to Ayatollah Kashani Orthopaedic clinic in Shahrekord, from April 2007 to April 2008 aged between 45 to 70 years old. Diagnosis of osteoarthritis and osteoporosis were based on history, physical examination, knee joint radiographs and Bone Mineral Densitometery (BMD). Inclusion criteria were: age between 45 to 70 years old, knee osteoarthritis, no history of metabolic diseases affecting bone and cartilage, such as gout, no previous history of trauma, ligamentous injury, absence of vascular disease and diabetes, signing informed consent by participants in the study.

Exclusion criteria were: Irregular use of drugs during the study period, not taking part in regular follow-up, not satisfied to participate in the study, existence of any metabolic diseases affecting bone and cartilage, existence of vascular diseases and previous history of trauma, Contraindications to use certain drugs and existence of hypersensivity in patients. Sampling was done in simple convenience method and assignments of each group were done in a randomized method based on random number table to Group I (combination of glucosamine (500mg TDS) and alendronate (70mg weekly)for 12weeks.) or Group II (glucosamine alone(500mg TDS)) for 12weeks.

Group I: Were given a combination of alendronate and glucosamine for 12 weeks.

Group II: Were given glucosamine alone for 12 weeks. For both groups 500 mg of calcium and 400 IU vitamin D was administered daily.

There are several treatments for osteoarthritis, including the use of glucosamine and since most of the patients had evidence of osteoporosis based on studies, treatment with alendronate appeared to be useful in the patients.

Oral tablet of glucosamine was administered three times a day and 70 mg oral tablet of alendronate was administered once a week. History, physical examination and radiographs of the knee in the first week were taken and then the patient’s symptoms were checked and signs were evaluated in the third, sixth and twelfth weeks of treatment. After data collection by check list they were recorded in Western Ontario and McMaster Universities Arthritis Index (WOMAC) questionnaire and severity of patients’ arthritis was measured by it. The questionnaire measured the severity of osteoarthritis based on the total scores of knee pain, stiffness and physical activity levels. Diagnosis was done by orthopaedic surgeon and evaluation of treatment results was done by another physician.

### 3.1. Statistical Analysis

After data collection SPSS16 software was employed to analyze the data through descriptive statistical tests including frequency, mean and standard deviation and comparison by t-test, X2 and ANOVA and repeated measurement statistical tests.

## 4. Results

Age range was 45-70 years old, with the mean 60.9 ± 9.9 years old. Independent t-test did not show a significant difference in the gender of the patients in both groups (P > 0.05). 95 patients (73.1%) were male and 35(26. 9%) were female. Chi-square test did not show a significant difference in the gender of the patients in both groups (P > 0.05). 35 patients (26.9 %) were overweight and distribution ratio was similar in both groups (P > 0.05). 90 (69.2%) patients had high level activity of knee and distribution ratio was similar in both groups (P > 0.05). Results of Pain intensity after 1, 3, 6 and 12 weeks after treatment are indicated in Table 1.

Reduction of pain intensity during the study was similar in both groups and there was no significant difference between the two groups (P > 0.05), (Table 1). The two groups had different levels of stiffness in the sixth and twelfth weeks after treatment and in group II (treated by glucosamine alone) level of stiffness was more than group I (treated by glucosamine and alendronate) and there were significant differences in both groups (P < 0.05). Level of knee stiffness during the study at 1, 3, 6 and 12 weeks after treatment are indicated in Table 2.

**Figure 1:**
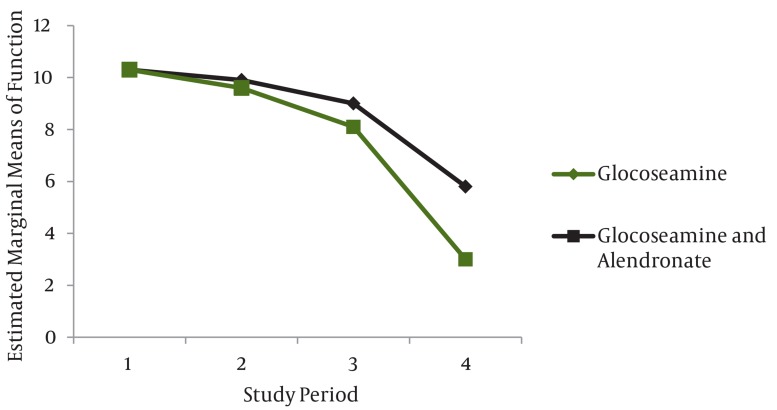
Function Results in the two Groups Treated with Alendronate and Glucoseamine, and Glucoseamine Alone, During the Study Period Based onWOMAC Index

**Figure 2:**
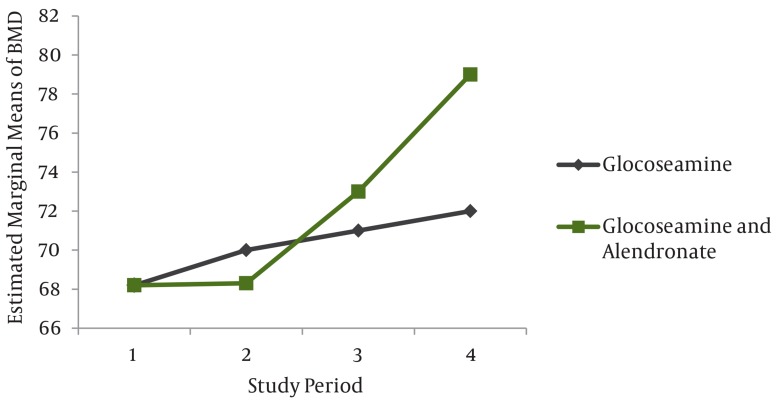
Evaluation of Percentage Corrected Bone Mineral Density (BMD) in the Group I and Group II During the Study Period

Level of knee activity during the study at 1, 3, 6 and 12 weeks after treatment are indicated in Table 3. ANOVA and repeated measurement tests showed that there was significant difference and increase in level of knee activity in both groups (P < 0.05) the group treated by glucosamine alone(group II) had a lower knee activity in the final stages of study (P < 0.05) (Figure 1). The results of corrected BMD in percentage in both groups during the study are shown in Table 4 and Figure 2. Repeated measurement test demonstrated significant increase in BMD during the study period (P < 0.05), and also showed that the rate of BMD increase was not the same in the groups, and that it was more in group I than group II (P < 0.05) (Table 4 and Figure 2).

**Table 1.** Pain Levels in the Group I and Group II During the Study Period.

	1 a	2 a	3 a	4 a	*P* Value
1th week					0.68
Group I b	-	1	13	51	
Group II b	-	2	10	53	
3th week					0.82
Group I	-	2	19	44	
Group II	-	3	21	41	
6th week					0.14
Group I	-	14	35	16	
Group II	2	19	36	8	
12th week					0.12
Group I	31	29	3	2	
Group II	22	32	10	1	

^a^Pain Intensity

^b^Group I received alendronate and glucosamine. Group II received glucosamine alone

**Table 2.** The Amount of Stiffness in the I and Group II during the Study Period Based on Western Ontario and McMaster Universities Arthritis Index (WOMAC) Index

	0 a	1 a	2 a	P value
1th week				1
Group I b	-	5	50	
Group II b	-	60	60	
3th week				0.07
Group I	1	37	27	
Group II	-	26	39	
6th week				0.01
Group I	5	44	16	
Group II	-	40	25	
12th week				> 0.01
Group I	32	28	5	
Group II	9	42	14	

^a^Pain Intensity

^b^Group I received alendronate and glucosamine. Group II received glucosamine alone

**Table 3.** The Function Results or Level of Activity in the Group I and Group II During the Study Period Based on WOMAC Index

Time	Group I a, Mean ± SD	Group II a, Mean ± SD
1th week	10/4 ± 3/1	10/4 ± 3/2
3th week	9/6 ± 3/0	9/4 ± 3/0
6th week	8/8 ± 2/3	7/9 ± 2/6
12th week	5/4 ± 2/1	2/9 ± 1/4

^3^Group I received alendronate and glucosamine. Group II received glucosamine alone

**Table 4.** Results of Percentage Corrected Bone Mineral Density (BMD) in Improving BMD in the Group I and Group II During the Study Period

	Group I a			Group II a		
	**Min**	**Max**	**Mean ± SD**	**Min**	**Max**	**Mean ± SD**
1th week	65	73	68.5 ± 1.8	65	73	68.5 ± 1.8
3th week	65	73	68.6 ± 1.8	66	74	70.2 ± 1.7
6th week	68	80	73.2 ± 2.8	68	76	71.4 ± 1.7
12th week	70	89	79.5 ± 2.8	68	79	73.5 ± 2.1

^a^Group I received alendronate and glucosamine. Group II received glucosamine alone

## 5. Discussion

The mean age, weight, activity level with knee, and gender distribution of patients in both groups were similar and the differences were not statistically significant (P > 0.05). Efficacy of the treatment in group I (combination of glucosamine and alendronate) and group II (glucosamine alone) regarding the patients` pain intensity level showed that after 12 weeks of treatment, patients pain had reduced in both groups similarly and there was not a statistically significant difference in both groups (P > 0.05). Several studies have found that administration of glucosamine has had a role in reducing pain in patients with osteoarthritis of the knee joint.

Mc Alindon et al. found improvement of pain at rest, during standing and exercise, limited activities and passive motion, after 50 days administration of glucosamine in patients with osteoarthritis (5). The results of this study are similar to that of the current study but the period of his study was shorter than that of the current study. Cibere found knee joint osteoarthritis pain reduction after administration of glucosamine and this reduction was greater than placebo (6). This result is similar to that of the current study but in the current study there was no placebo drug. Clegg also found pain reduction after administration of glucosamine compared with placebo in patients with osteoarthritis (7) this result is similar to that of the current study but in the current study there was no placebo drug. Qui`s study also showed pain reduction in patients with knee osteoarthritis after administration of glucosamine compared to Ibuprofen and showed that glucosamine was better than Ibuprofen and also the tolerance was better than it (8) these results are similar to that of the current study but in the current study no anti-inflammatory drugs were used. Carbon`s study also showed that alendronate reduced the intensity of knee pain in patients with osteoarthritis (9). Hayami also showed that alendronate reduced the incidence of osteophytes formation that was a known mechanism for joint pain, and though it could indirectly reduce the pain (10). Results of these studies are found to be similar with those of the current study. So glucosamine can still play a role in reducing joint pain in knee osteoarthritis and it is an important treatment option. Even if glucosamine does not reverse the cartilage changes, its pain reduction mechanism can compete with NSAID’s and can have beneficial effects on life quality of patients with osteoarthritis.

Some causes of pain in osteoarthritis are inflammation of the synovium and joint capsule. Glucosamine like an anti-inflammatory drug suppresses the two mentioned mechanisms and prevents pain stimulation and pain will decrease over the 12-week course of treatment (11). Bone fractures under the cartilage can cause joint pain in osteoarthritis, in this case the administration of alendronate can prevent bone restoration, can increase bone strength to prevent such fractures, and can reduce pain in some patients. Analysis of articular cartilage in the osteoarthritic knee joint showed that unequal distribution of force on the ligaments, muscles and skin, causing strain and spasm , can lead to knee pain (10, 12). The role of glucosamine administration on cartilage and chondrocyte proliferation and production of collagen is preventing pain by mentioned mechanism and leading to better function of joint. Osteophytes formation is often a result of disproportionate force on subchondral bone, osteophytes irritate nerve endings in subchondral bone and can cause pain. The glucosamine administration can repair cartilage, prevent the creation of osteophytes, and eliminate one of the pain creating mechanisms. One of the alendronate roles in preventing and reducing joint pain is inhibiting osteophytes formation. A reason that the two groups did not differ in pain reduction was due to the fact that musculoskeletal pain is a common side effect of alendronate, while alendronate can eliminate the pain mechanism, it can be a pain producer agent. The stiffness decreased in both groups during treatment period but this reduction was significantly greater in the combined treatment group (group I). Some studies have pointed out the role of glucosamine in reducing joint inflammation (12-14). If it is known that inflammation causes joint stiffness, glucosamin, by reducing inflammation, can also reduce stiffness. The addition of alendronate to glucosamine leads to reduction of joint stiffness more than glucosamine alone, and this is due to strengthening subchondral bone to prevent fractures and osteophytes formation, and both of these factors can lead to muscle spasms, inflammation of the ligaments, joint inflammation, and stiffness and spasms, that after administration of alendronate, reduce and joint movements will be easier and softer. Joint level activity during 12 weeks period of study showed a significant increase of activity in both groups (P < 0.05) that this difference was significant in later stages of the study(12th week) in both groups, reduction of knee stiffness in group II( receiving the glucosamine alone) was less than group I (alendronate and glucosamine).

The role of glucosamine that improves joint function has been proved in various studies (5-8) but the role of alendronate on joint function in osteoarthritic patients, has not been studied. This study demonstrated the beneficial role of combination therapy of alendronate with glucosamine in improving joint function that can be due to below findings. One of the limiting joint activity levels is pain (9, 10, 15). Alendronate can reduce joint pain, so addition of glucosamine to it has a significant role in improving joint function. As mentioned, combination of glucosamine and alendronate lead to more reduction of joint stiffness and thus more improvement of joint function, so further improvement in group I that is treated with combination of glucosamine and alendronate is expected. Repeated measurement test showed a significant increase in BMD during the study, it also showed that the rate of BMD increase was not the same in both groups and in the combination group treatment differences was significant (P < 0.05). These differences were due to histological effects caused by glucosamine on cartilage and prevention of absorption effect of alendronate on bone density. As alendronate is a drug of choice in osteoporosis treatment which makes changes in bone tissue, it leads to less osteophytes formation and strengthens the subchondral bone and prevents osteoporosis, it can be concluded that increase of BMD is higher in the combination treatment of gucosamine and alendronate, which is very useful. Combination treatment of glucosamine and alendronate was more effective than glucosamine alone in improvement of stiffness, function, radiographic and BMD changes of knee joint osteoarthritis but regarding joint pain reduction, these two treatments were similar.

### 5.1. Recommendation

1 - Glucosamine in patients with osteoarthritis as one of the first line treatments should be considered, because the current treatments such as NSAID’S in the elderly patients are associated with numerous complications.

2 -The combination treatment of alendronate and glucosamine, especially in menopausal women who have osteoporosis and are more likely to develop osteoarthritis, will lead to better results, and it is recommended as an effective treatment.

3 - While taking these medications, physical therapy and rehabilitation can be considered which lead to inflammation reduction in the joints, and increase activity level. 
